# Cerebral circulation and ischemia: How to monitor it during surgery?

**Published:** 2015

**Authors:** Kun Liu, Hong Liu, Wei Dong Gao, Lingzhong Meng

**Affiliations:** 1 Nanhua University; 2 University of California Davis; 3 Johns Hopkins University; 4 University of California San Francisco

The anesthesiologists from both the United States and China had an open and engaging discussion on intraoperative monitoring of cerebral perfusion and ischemia at the social media – WeChat International Chinese Academy of Anesthesiology (ICAA, www.icaahq.org) group. This discussion is presented here to showcase the role the modern social media plays in enhancing the academic exchange and learning experience on the international stage. Some minor edits were performed for clarity purposes.

## Kun Liu (University of Sounth China)

I have a question and would like to have your opinion. Is anesthesia depth monitoring clinically meaningful in patients undergoing neurological surgeries such as traumatic brain injury, intracranial hemorrhage, brain tumor, etc.?

## Lingzhong Meng (University of California San Francisco, USA)

Dr. Liu, whether deep anesthesia such as burst-suppression is neuroprotective is still unclear. We routinely use Electroencephalography (EEG) monitoring during aneurysm clipping. The surgeon normally request burst suppression. For brain tumor cases, we do not normally monitor bispectral index (BIS), etc. However, the surgeon may perform cortical and subcortical mapping if the tumor is adjacent to eloquent brain in order to maximize the extent of resection. For brain trauma, I personally do not think anesthesia depth monitoring is useful. What matters in traumatic injury cases is to decrease intracranial pressure (ICP) and maintain cerebral perfusion and oxygenation.

## Yongqing Guo (Shanxi Provincial Hospital, China)

Dr. Meng, what types of anesthesia monitoring are used during neurosurgical procedures in the United States?

## Lingzhong Meng

Dr. Guo, how to monitor neurosurgical patients is institution and surgeon dependent. Now, as anesthesiologists, we would like to monitor intravascular volume status, tissue perfusion, etc. This is important because mannitol that is almost routinely given to patients causes large urine output and volume depletion as a consequence. Also, hyperventilation if adopted can cause vasoconstriction, especially the brain, i.e., cerebral vasoconstriction.

## Wenqi Huang (Sun Yat-Sun University, China)

I have a different school of thought. EEG monitoring is very valuable in anesthetized patients. The target of general anesthesia is the brain. The electrophysiological activity of the brain is what the anesthesiologists can rely on, especially when the clinical picture is confusing. The patient complains dizziness when the blood pressure is decreased by epidural or spinal anesthesia. The awake elderly patient will complain dizziness and sickness if the systolic blood pressure is less than 100 mmHg. The patient who is under general anesthesia is unable to tell his/her feeling. The individualized index of cerebral perfusion is the percentage of the Delta wave on EEG tracing that can tell when the blood pressure cannot be further lowered. Cerebral perfusion pressure (CPP) equals mean arterial pressure minus ICP. CPP needs to be maintained above 70 mmHg. It cannot be less than 55 mmHg in patients with traumatic brain injury. Therefore, we should be able to tell how the mean arterial pressure should be maintained under various scenarios. As anesthesiologists, we did not pay the needed attention to hypotension. Therefore, I recommend EEG monitoring for blood pressure care.

## Lingzhong Meng

Dr. Huang, thank you for sharing your thought! Where is the relationship between blood pressure and EEG coming from? Would you please share with us the evidence, not just personal opinion?

## Wei Dong Gao (Johns Hopkins University, USA)

Dr. Huang and Dr. Meng, what are the effects of anesthetic agents on EEG? Is cerebral tissue oxygen saturation (SctO_2_) monitoring more valuable?

## Lingzhong Meng

Dr. Gao and Dr. Huang, in our institution, we do not use EEG monitoring to guide blood pressure management. Neither do we use BIS for blood pressure care. SctO_2_ has the potential to guide blood pressure care. However, the relationship between blood pressure and SctO_2_ is complex. A currently popular point of view is that cardiac output is probably more important for SctO_2_ (than blood pressure). Therefore, non-invasive cardiac output monitoring is probably more valuable to tell how the tissue is perfused. A fixed number of cerebral perfusion pressure (CPP) cannot be used to tell if the brain is adequately perfused. A mean arterial pressure of 80 mmHg may not be adequate for cerebral perfusion in a hypertensive patient [1]. SctO_2_ monitoring is a valuable surrogate of cerebral blood flow. In addition, the brain is a vital and prioritized organ. Even if it is perfused okay, the other organs like the gut and the muscle may not (be well perfused). Tissue hypoxia or desaturation, even if not the brain, may be one of the root causes of adverse outcomes.

## Wei Dong Gao

Dr. Meng, the relationship between blood pressure and SctO_2_ is indeed complex. It may vary at varying cerebral autoregulatory ranges. Therefore, caution is needed when using a “normal” value (or a fixed number) to guide blood pressure care. Relevant and recent studies are available, including the work by Dr. Hogue and Brady (from Johns Hopkins).

## Wenqi Huang

Dr. Meng, thank you for sharing your opinion. The cerebral blood flow is 50 ml/100g/min in adults. Slow wave appears on EEG monitoring when the cerebral blood flow is less than 20 ml/100g/min (please refer to Miller’s Anesthesia book chapter). There is a close relationship between CPP and blood pressure. This is similar to the change in ST segment on electrocardiogram monitoring that is used to monitor myocardial ischemia. In patients with acute ischemic stroke, the treatment goal is to maintain blood pressure in addition to prompt thrombolysis.

## Jie Sun (Jiangsu Provicial Hospital, China)

Dr. Meng, when you say the cardiac output is likely more important to cerebral oxygenation (than blood pressure), do you suggest that a faster heart rate is less problematic to cerebral oxygenation even though the blood pressure is relatively low after cardiopulmonary bypass? Or, can you tell which physiological status (in terms of blood pressure, heart rate and cardiac output) is safer (for cerebral perfusion and oxygenation)? It seems that these newer findings do not agree well with the lower limit of cerebral autoregulation.

## Lingzhong Meng

Dr. Huang, your points of view are very unique. They are refreshing to me. I am not prepared to respond your queries at the moment. These are just friendly reminders. Critical thinking is needed during reading. Different issues or questions need to be treated differently. A conclusion can be stated in one direction; however, it cannot be applied in clinical practice in a reverse direction (because the cause-effect relationship is changed). Textbooks cannot be used without distinction. Dr. Miller’s office (the editor of the most popular anesthesia textbook) is 50 meters away from my desk. Views can be discussed, but cannot be imposed. Otherwise, it causes more harm than good. It is well known that CPP depends on blood pressure. This is the knowledge a medical student who wants to be a pediatrician in the future knows. Nonetheless, I really enjoyed the exchanges with you. Thank you for your courage and candor.

Dr. Sun, thank you for your interest. We are drafting a review to address this issue at the moment. We believe that tissue perfusion and oxygenation are probably better when cardiac output is ideal even though blood pressure is low compared with that when cardiac output is low while blood pressure high.

## Jie Sun

Dr. Meng, thank you! The increased incidence of stroke in the POISE study may have something to do with the reduced cardiac output in addition to hypotension [2].

## Wenqi Huang

Dr. Sun, the mean arterial pressure should be maintained at 60~80 mmHg when the temperature is 34°~35° during cardiopulmonary bypass. The incidence of brain injury is increased if the blood pressure is fluctuating during euthermic (normothermic) cardiopulmonary bypass that emphasizes the importance of intraoperative blood pressure care. However, the brain is not injured when its perfusion is stopped for 30~40 minutes as long as the core temperature is decreased to be less than 17° when the cerebral metabolic rate (represented by cerebral metabolic rate of oxygen (CMRO_2_) and Q10) is 10 times less than euthermia (normothermia). However, irreversible brain injury occurs if the cerebral blood flow is less than 12~15 ml/100g/min and the body temperature is normal. The EEG monitoring during surgery provides valuable information for blood pressure and ventilation management.

Dr. Meng, the cerebral blood flow depends on the age. It is 97 ml/100g/min in pediatric patients less than 6 months old, 85 ml/100g/min in those 3–6 years old, and 70 ml/100g/min in those 7–12 years old. Multiple factors can cause inadequate cerebral perfusion; however, hypotension is an important one. The blood pressure management needs to be individualized. The baseline blood pressure needs to be maintained. I suggest to use EEG monitoring to facilitate blood pressure care.

## Hailong Dong (Xijing Hospital, China)

Dr. Meng and Dr. Huang, I am inspired by your discussion. Blood pressure is not the only variable that determines cerebral perfusion, neither is it the foundation of cerebral oxygenation. Whether EEG monitoring can independently tell how the brain is perfused and oxygenated is still controversial. This is suggested by the “Triple low” study; however, convincing evidence is needed.

## Tiegang Wei (Mudanjiang Cancer Hospital, China)

Dr. Dong, I believe, even though EEG does not measure cerebral perfusion directly, the relationship between the change in EEG monitoring (value?) and the adequacy of cerebral perfusion is trustworthy.

## Lingzhong Meng

Special thanks to Dr. Huang and Dr. Dong! The opinions from both of you are making this open discussion more meaningful. Cerebral blood flow stays relatively stable when the blood pressure fluctuates, as long as the cerebral perfusion pressure is higher than the lower limit, according to cerebral autoregulation. The cerebral perfusion pressure-cerebral blood flow relationship is not passive, meaning that, even though the blood pressure is decreased, the cerebral circulation stays the same. The challenge during surgery is to know if the cerebral autoregulation is still functional and where the lower limit is. We definitely need to maintain the CPP above the lower limit. However, this lower limit may change during surgery [1].

## Wei Dong Gao

Dr. Huang, during cardiopulmonary bypass, it is well accepted that mean blood pressure be maintained at 60–80 mmHg and this range has been used in most hospitals. But, this range is largely based on the well-known cerebral autoregulation curve. Recent studies have shown that the range of cerebral autoregulation, especially in patients with hypertension and advanced age, is different from person to person. Therefore, post-cardiopulmonary bypass brain complications are common, likely due to the inaccurate blood pressure management during bypass. The most rigorous and scientific way to do is to find the cerebral autoregulation range of the patient and manage blood pressure based on this range. Unfortunately, no hospitals are doing it (monitoring autoregulatory range) at present. This is why it is necessary to conduct clinical studies in perioperative monitoring of cerebral oxygenation during open-heart surgery. The most straightforward way to find cerebral autoregulation range is measure blood pressure and cerebral blood flow directly, but direct measurement of cerebral blood flow is not practical during surgery. This is why Drs. Brady and Hogue discovered using SctO_2_ based on near-infrared spectroscopy (NIRS) as a surrogate of cerebral blood flow.

## Xin Kuang (University of South China, China)

I always see perfusion pressure decreased to 20–30 mmHg as soon as the cardiopulmonary bypass initiates. Please advise !

## Wei Dong Gao

This range of blood pressure is too low. What about postoperative complications?

## Xin Ye (United Family Healthcare Beijing, China)

I had a few patients with severe cerebrovascular stenotic lesions when I was working at Xuanwu Hospital. I used the mean blood pressure when the patients were hospitalized (before surgery), in addition to the symptoms of cerebral ischemia (before surgery), as the blood pressure management goal during cardiopulmonary bypass.

## Jie Sun

I agree. The emphasis on blood pressure maintenance can leads to overuse of vasoactive drugs, which is good for the brain but may be bad for the heart.

## Wei Dong Gao

This is a more practical approach.

## Xin Kuang

Postoperative cognitive impairment increases as the mechanical ventilation time increases!

## Lingzhong Meng

Dr. Wei, here is the problem. I agree that inadequate cerebral perfusion leads to EEG change. However, a change in EEG does not necessarily mean the cerebral perfusion is becoming inadequate. For example, the change in anesthesia depth is probably the most common cause of EEG change. Low cerebral perfusion is just one of the potential causes of an EEG change. In addition, the cause of insufficient cerebral perfusion is also multifactorial. For example, both a decrease in cardiac output and hypocapnia secondary to hyperventilation can result in a decrease in cerebral blood flow. Interestingly, it was observed that SctO_2_ was decreased when the blood pressure increased (by phenylephrine) [3]. Our recent work failed in showing a direct relationship between blood pressure and SctO_2_ (and the tissue oxygenation monitoring on the leg). One of the potential explanations for this observation is cerebral autoregulation.

## Wei Dong Gao

Both brain and heart should be protected. It is therefore emphasized to employ a scientific and reliable monitoring method during (open heart surgery). I really hope colleagues in China to conduct more research using NIRS, because there is a lot of potential of conducting clinical studies in China.

## Hongda Cai (Fujian Medical University, China)

Dr. Meng, if a patient’s baseline blood pressure (mean) is 90 mmHg and assuming the autoregulatory range is 60–160 mmHg, should his blood pressure (mean) be maintained at 60 mmHg instead of his baseline (90 mmHg)? Will maintaining his blood pressure at baseline (90 mmHg) facilitate regional blood flow and metabolic substrate exchange?

## Lingzhong Meng

Dr. Cai, this is a great question. Dr. Gao, I meant both cerebral and muscular oxygenation. The manuscript was submitted recently.

## Hongda Cai

Perfusion pressure does affect cerebral blood flow. However, these are two different entities and their relationship should be treated carefully.

## Wei Dong Gao

Dr. Cai, if the autoregulation range is accurate, it means cerebral blood flow does not change as blood pressure changes. Local metabolism should not be expected to change as well.

## Wei Dong Gao

Dr. Meng, it (the results of your recent study) makes sense.

## Hongda Cai

The major problem (in daily practice) is that we do not know the autoregulatory range (under various conditions). Therefore, we use the strategy to keep the intraoperative blood pressure at the patient’s baseline. However, the use of vasopressor can lead to a decreased cardiac output secondary to decreased heart rate and increased afterload (if using pure α-agonist) even though the blood pressure is increased to the baseline level.

## Lingzhong Meng

Dr. Cai and Dr. Gao, different perfusion pressure leads to different cerebral vasomotor tones within the autoregulatory range because, in order to keep the flow stable when the perfusion pressure is changed, the flow resistance (vasomotor tone) needs to change accordingly. Therefore, I speculate this (different vasomotor tone under different perfusion pressure) may lead to different outcome. Maintaining the intraoperative blood pressure around the baseline is a great guess (for better outcome).

## Wei Dong Gao

Dr. Cai, this depends on the situation. If blood pressure is low enough to decrease blood supply to the heart, increasing blood pressure can increase cardiac contractility and cardiac output. Blood supply to cardiac tissue mostly occurs during diastole.

## Tiegang Wei

Dr. Meng, here is a thought that may deserve attention. The surgical stimulation and anesthetic depth are relative constant during cardiac surgery; therefore, I believe the change in EEG monitoring is more meaningful (during cardiac surgery).

## Hongda Cai

Will the outcome be different between two different managements in which the blood pressure (mean) is maintained at 90 and 60 mmHg, respectively? The cerebral blood flow should be the same if the autoregulation is functional.

## Xin Ye (United Family Healthcare Beijing, China)

We had studies on how the monitoring of the middle cerebral artery flow velocity using transcranial Doppler affects postoperative outcome.

## Wei Dong Gao

Dr. Ye, this is essentially the same as monitoring cerebral oxygenation.

## Xin Ye

Yes. However, this is not as sensitive as EEG. Unfortunately, there are not many hospitals in China that use intraoperative EEG monitoring.

## Wei Dong Gao

One should realize that SctO_2_ monitoring and EEG monitoring are different. Based on current evidence, monitoring cerebral oxygenation is relatively clinically more significant because it seems associated with a better clinical outcome. Unless someone can show that EEG monitoring can also lead to a beneficial outcome: i.e., postoperative (especially open heart surgery) neurological complications.

## Lingzhong Meng

We routinely monitor EEG (during aneurysm clipping). However, we use it to guide the care of anesthesia depth (burst-suppression), not cerebral perfusion, and for sure not blood pressure.

## Xin Ye

We had used cerebral oximetry. My personal experience is that it only measures the hemisphere and does not precisely measure the brain region that has the potential of ischemia. EEG has better precision by monitoring the brain area with ischemic risk secondary to, for example, cerebrovascular stenotic lesions.

## Hong Liu (University of California Davis, USA)

I am fascinated by the discussion. To my knowledge, the current blood pressure range for cerebral blood flow autoregulation in the textbook was reported in late 1950s. This value may not be very accurate. However, as the rule of thumb, it is important to keep the blood pressure within the 20–30% of the baseline value. This raised another question, what is the baseline blood pressure? Is the blood pressure we obtained right before surgery is the baseline blood pressure or the blood pressure recorded in the patient medical record 3 months ago is the baseline blood pressure? Is EEG a good indicator for intraoperative cerebral perfusion? I personally do not think EEG is, because it could be affected by too many factors, such as: anesthetic agents just to name one. Because there is no single good cerebral perfusion monitoring method available, the cerebral oxygen saturation is the best alternative that is currently available.

## Lingzhong Meng

Dr. Ye, in order to achieve the precision you mentioned, how many needles do you need to put on the scalp? There is no gold standard to diagnose cerebral ischemia. Otherwise, we will have to go to the court if, as a doctor, you do not use the gold standard for patient care (monitoring cerebral ischemia in risky cases).

## Hong Liu

Dr. Kuang, there is a transit decrease in perfusion pressure when the cardiopulmonary bypass was initiated. It is important to communicate with the perfusionist when it occurs. It is necessary to use vasoactive medications if the perfusion pressure is still low and the cerebral oxygen saturation below the baseline.

## Tiegang Wei

The BIS value changes when the cerebral perfusion pressure changes during cardiopulmonary bypass. This can be an important warning sign. However, large scale studies are needed to determine its threshold.

## Renyu Liu (University of Pennsylvania, USA)

Cerebral oximetry was applied in cardiopulmonary bypass surgery when it was first introduced to clinical practice in 1990s. However, it represents regional cerebral oxygenation that is determined by the balance between cerebral oxygen consumption and supply, and is affected by various factors. It does not represent cerebral blood flow or cerebral function. It cannot be used reliably to monitor regional cerebral ischemia. For example, SctO_2_ may decrease even if the cerebral perfusion is increased or stable when the core temperature in increased at the end of cardiopulmonary bypass.

## Tiegang Wei

It (SctO_2_) is more informative if the relevant factors (determining) are kept stable.

## Renyu Liu

Agree. It (SctO_2_) is the “trend of change” that is meaningful.

## Xin Kuang

Dr. Liu, thank you! What is the mechanism of the transient perfusion pressure decrease? Another question, can BIS monitoring tell the depth of anesthesia? Can it guarantee that the intraoperative awareness will not occur? I noticed that the dose of the anesthetic agents used in the United States is much smaller than that used in China. I was worried about the awareness.

## Hong Liu

Because the area (the forehead) is limited, I usually only use cerebral oxygen saturation monitoring during cardiac surgery. I actually never used BIS monitoring in cardiac surgical patients.

## Zhongling Xu (Jiangsu municipal Hospital, China)

Intraoperative awareness should not occur if enough midazolam plus inhalational anesthetics are used.

## Jie Sun

I agree with Dr. Liu. EEG and BIS may be able to tell cerebral perfusion and metabolism depending on the aspects we are referring to. However, it may be proven unreliable by future research. That is why the evidence-based medicine is forever changing.

## Tiegang Wei

I agree with Dr. Sun. There currently no monitoring or management strategy that can prevent intra-operative awareness.

## Hong Liu

I practice enhanced recovery after surgery (ERAS) protocol; therefore, I rarely use midazolam. I have not encountered any case with intraoperative awareness yet.

## Jie Zhou (Brigham and Women’s Hospital, USA)

Awareness is a confusing concept. It is awareness in English. It is often confused with recall.

## Jie Sun

I never saw a case of awareness even though the textbook says it has high incidence. This may due to the fact that, when I decrease the anesthesia depth during hypotension, I have a bottom line in addition to referring to the monitoring data. The management of hypotension is made much easier with the help of transesophageal echocardiogram (TEE).

## Xin Ye

I had an idea before. The lowest tolerable blood pressure in patients undergoing carotid endarterectomy can be determined by gradually decreasing the blood pressure in a controlled manner until the symptoms of cerebral ischemia occur. At the same time, we monitor transcranial Doppler. This approach (maintaining a lower blood pressure) can increase cerebral oxygenation, decrease cerebral metabolic activity (by general anesthesia), and decrease cardiac work load (by avoiding vasopressor?).

## Tiegang Wei

The key is to precisely tell when the brain is undergoing ischemia.

## Lingzhong Meng

Dr. Ye, the idea you just mentioned is risky! A study from Columbia University (published in Neurosurgery) showed that the outcome is better if the intraoperative blood pressure is maintained 20% above the patients’ baseline value during carotid endarterectomy. It is crucial not to let the blood pressure decrease in this patient population.

## Xin Ye

I meant to do it before surgery. Yes, the protocol did not go through due to the concern of severe adverse outcome.

## Lingzhong Meng

There are many published studies showing the relationship between intraoperative SctO_2_ monitoring and postoperative outcomes [4]. One should remember though, we routinely monitor arterial blood hemoglobin oxygen saturation using pulse oximetry (SpO_2_, not SctO_2_); however, there are no convincing studies showing that it is beneficial to outcomes. We believe SctO_2_ monitors a crucial cerebral circulation regulation mechanism that is neurovascular coupling [5]. Neurovascular coupling regulates cerebral blood flow based on the metabolic demand. When the cerebral metabolic activity is increased, this mechanism increases the cerebral blood flow (supply) accordingly in order to meet the increased demand, and *vice versa*. SctO_2_ is essentially determined by the balance between cerebral oxygen consumption and supply. Therefore, we argue that it assesses the functional status of neurovascular coupling. If the mechanism of neurovascular coupling is intact during anesthesia, the cerebral perfusion and oxygenation will be regulated based on the cerebral metabolic activity. This would be great! Neurovascular coupling is different to cerebral autoregulation. They are both important mechanisms regulating cerebral hemodynamics. These distinctive mechanisms clearly demonstrate the importance of the brain as a vital organ.

## Jie Sun

Dr. Meng, this is indeed complicated. It is complex when trying to understand the effect of vasopressor (on cerebral oxygenation) when there are both change in blood pressure and consequential change in cardiac output. In addition, the outcome may differ between two management strategies that use vasoactive drugs during hypotension and normotension, respectively. The BJA paper showed vasopressor (like norepinephrine and phenylephrine) can decrease cardiac output during euvolemia; however, it may guarantee the blood supply to the brain and hear during hypovolemia via vasoconstriction of peripheral organs [3].

## Wenqi Huang

Dr. Meng, the cerebral oxygen supply equals to hemoglobin times 1.36 times cerebral blood flow. SctO_2_ represents the dissolved oxygen content in the blood (not the oxygen carried by hemoglobin, however, please correct me if I am wrong); therefore, cerebral blood flow is a better indicator of cerebral metabolic demand. Inhaled anesthetics, when it is used in a large dose, can alter cerebral autoregulation, that means that the cerebral blood flow will not stay stable at the perfusion pressure range of 60~150 mmHg and an increase in blood pressure can lead to an increase in cerebral blood flow. In addition, the main problem during stroke is not vasospasm, but the flow stoppage caused by the embolus coming from the broken atherosclerotic plaques. Briefly, we should first pay attention to blood pressure (it only takes 15 seconds to see the symptoms of cerebral ischemia) when intraoperative EEG is changed (decreased), followed by adjustment of anesthetic dose. A deep EEG if it is not caused by hypotension is featured by slow eye movement sleep that helps brain resting.

## Buwei Yu (Shanghai Ruijin Hospital, China)

The difference between the West and the East is typically demonstrated by the exploration of the relationship between two different entities. The West insists to use study to test the hypothesis. They first measure these two entities, followed by statistical analysis and p-value to either accept or refute the hypothesis. This is an example of modern science based on statistics. In comparison, the East does it differently by using logical philosophy. This is one of the examples. If the cerebral oxygen consumption is constant, the cerebral oxygenation (SctO_2_) should correlate with the cerebral blood flow because the oxygen is carried by the blood. Why bothered to study it? It is simply self-evidenced. This may be one of the key reasons that the scientists in China are more difficult to obtain Nobel Prize than these in the West.

## Lingzhong Meng

Many thanks to Dr. Yu for his participation in this discussion from a philosophical point of view. Philosophy is the flower of wisdom. Philosophical thinking is crucial for academic advancement. They supplement and benefit each other. After all, we all benefit from this open and wide-range discussion and would like to thank those who had participated in this forum!

## Summary

The brain constitutes only 2% of the body weight; however, it disproportionately receives 12% of the cardiac output [6]. The wellbeing of the brain or the survival of neurons and glia relies on continuous and adequate cerebral blood flow to supply the metabolic substrates needed and washout the waste being produced. However, cerebral ischemia and hypoxia can occur during surgery due to either the flow-restrictive cerebrovascular lesions and/or the hemodynamic disturbances related to surgery and/or anesthesia. Monitoring cerebral perfusion and oxygenation during surgeries that harbor high risk of causing cerebral ischemia can facilitate the timely detection of cerebral ischemia and hypoxia and trigger prompt interventions to reverse these hazardous physiological processes. It became clear that there is no such a gold standard of intraoperative cerebral perfusion and ischemia monitoring even though a multitude of monitoring modalities are currently available. A systematic review on this important issue is needed in order to better guide clinical practice and identify the directions for future research.

## References

[1] Meng L, Gelb AW (2015). Regulation of cerebral autoregulation by carbon dioxide. Anesthesiology.

[2] Devereaux PJ, Yang H, Yusuf S, Guyatt G, Leslie K, Villar JC, Xavier D, Chrolavicius S, Greenspan L, Pogue J, Pais P, Liu L, Xu S, Málaga G, Avezum A, Chan M, Montori VM, Jacka M, Choi P, POISE Study Group (2008). Effects of extended-release metoprolol succinate in patients undergoing non-cardiac surgery (POISE trial): a randomised controlled trial. Lancet.

[3] Meng L, Cannesson M, Alexander BS, Yu Z, Kain ZN, Cerussi AE, Tromberg BJ, Mantulin WW (2011). Effect of phenylephrine and ephedrine bolus treatment on cerebral oxygenation in anaesthetized patients. Br J Anaesth.

[4] Nielsen HB (2014). Systematic review of near-infrared spectroscopy determined cerebral oxygenation during non-cardiac surgery. Front Physiol.

[5] Meng L, Gelb AW, McDonagh DL (2013). Changes in cerebral tissue oxygen saturation during anaesthetic-induced hypotension: an interpretation based on neurovascular coupling and cerebral autoregulation. Anaesthesia.

[6] Williams LR, Leggett RW (1989). Reference values for resting blood flow to organs of man. Clin Phys Physiol Meas.

